# Towards a Behavioral-Matching Based Compilation of Synthetic Biology Functions

**DOI:** 10.1007/s10441-015-9265-9

**Published:** 2015-07-05

**Authors:** Adrien Basso-Blandin, Franck Delaplace

**Affiliations:** IBISC Laboratory, Evry University, Évry, France

**Keywords:** Domain specific language, Synthetic biology, Compilation

## Abstract

The field of synthetic biology is looking forward engineering framework for safely designing reliable *de-novo* biological functions. In this undertaking, Computer-Aided-Design (CAD) environments should play a central role for facilitating the design. Although, CAD environment is widely used to engineer artificial systems the application in synthetic biology is still in its infancy. In this article we address the problem of the design of a high level language which at the core of CAD environment. More specifically the **Gubs** (Genomic Unified Behavioural Specification) language is a specification language used to describe the *observations* of the expected behaviour. The compiler appropriately selects components such that the observation of the synthetic biological function resulting to their assembly complies to the programmed behaviour.

## Introduction

The design of safe and secure synthetic biological functions is a major challenge in synthetic biology. In this undertaking, computer-aided-design (CAD) environments should play a central role by providing the required features to engineer systems: specification, analysis, and tuning (Bilitchenko et al. 2011; Pedersen 2009; Umesh et al. 2010; Czar et al. 2009). Thus, increasing the complexity of synthetic biological devices with the design of *de-novo* synthetic genome as a long-term goal (Gibson et al. 2010) naturally leads to investigate the automatic conversion processes of the design specification into biological components like compilers for programming languages. In this context, high level programming language for synthetic biology is announced as a key milestone for the second wave of synthetic biology to overcome the complexity of synthetic system design (Purnick and Weiss 2009) by providing the ability for researchers to describe abstractly and concisely function while compiling it into a low level representation such as DNA sequences.

However, the nature of the medium—a living organism—requires to revisit the methods used in compilation to account its specificity. Indeed, although living organism is theoretically viewed as system (Kaneko 2006; Kitano 2002), its design notably differs from other engineering systems. Usually, the design in system engineering is mostly based on a reductionist approach consisting of a hierarchical composition of inter-operable and modular parts. In this methodological context, the functionalities referring to a given level are defined as an assembly of devices corresponding to the level immediately below. For example, this methodological framework architectures the design of computer network.

Although this framework is a standard for system design, the application in synthetic biology encounters some fundamental difficulties. The origin of these difficulties could be summarized by the fact that the design essentially consists in re-engineering a natural system resulting of a Darwinian selection that seemingly does not follow the afore mentioned principles in its ‘design’. In particular, the following characteristics must be accounted for the design and the compilation process:

*Structures with multiple functionalities* The design a biological system aims at devising functionalities that do not exist in the Nature. However, pointed out by Jacob (1981) as the consequence of the evolution, the biological components may support several functions leading to the absence of a one-to-one mapping between structures and functions. Thereby, a structure may encompass different functions. Thus, the design of a biological system can be roughly summarized as selecting a part of functionalities in components to form a device tiling the expected synthetic biological function (Stocker et al. 2003). Besides, The same functionality can be carried out by different biological structures. For instance the inhibition of the expression of the protein can be achieved either by an inhibitor or by RNA silencing mechanism.

*Interaction with the environment and incompleteness of the models* The interactions can be envisioned in twofold: by considering the environmental conditions characterizing a particular context for process triggering and as a possible source of disturbance possibly preventing the realization of the expected result. Although the progress made in the field of molecular biology, the complete understanding of an organism remains out of our knowledge, then limiting the scope of the control action on the organism.

To circumvent these difficulties, our proposal is to specify a biological function as “*a set of expected observations related to causal interactions*” and the compiler is then in charge of delivering the right assembly of biological components to obtain the expected observations. Informally, a program is here *an observation based query* described by the observations of causal relations. Hence, the programmer does not explicitly describe the process chain supporting a function but rather the expected observations of the designed function by emphasizing causal relations. The compiler then selects a set of components in a database such that their assembly complies to the program specification. Informally, the compilation process relies on a syntactical pattern matching such that the description of the selected components tile the description of the behaviour. Hence, a functional description of the behaviour is associated to each component and the union of the selected components behaviourally covers the programmed functionality. By contrast to the standard compilation process based on the definition of a morphism^1^ from syntax to objects of calculus underpinning the hierarchical assembly, the tiling is here global and orderless. Notice also, that only a piece of the functionality of a component could be used for this covering. Thus, the design gains in abstraction and in flexibility for component selection while accounting the multiple functionalities for a components and possible extensions.

In literature, researches on programming languages for synthetic biology are mainly focused on a structural description (Czar et al. 2009; Pedersen 2009; Bilitchenko et al. 2011) of components used to specify a well-formed genome sequences. By contrast, our proposal is focused on the function description postponing the biological components selection at compile phase, motivated by the facts that the size of the structural description significantly increases when the complexity of programmed systems increases. Besides as the same function can be carry out by different structures (*e.g.*, DNA sequences), the compiler may select a component amongst a set of functionally equivalent components in accordance to a context.

Beal et al. (2011) has developed a compiler (BioCompiler) translating amorphous programs written in Proto into DNA sequence. The biological components are assimilated to actuators and logical gates. Although the compilation scheme is function oriented it differs to **Gubs** on the following orientations: a **Gubs** program describes the observation of a biological system instead of an amorphous process, and the compilation scheme does not assimilate the component functionalities to logic gate but describe them by causal relations allowing more flexibility for the description. Hence, the components are not necessary atomic (*e.g.*, promoter) but correspond to a collection of already tested components with different scales.

In Rodrigo et al. (2012), the authors the design is based on the pathway analysis to find a way to produce specific biological compounds. In **Gubs**, the assembly is based on a tiling of the expected behaviour by the behaviour of the components.

In this article, we study a domain specific language **Gubs** and a compiler providing a framework for behavioural description of biological component while accounting the openness of such system. After introducing the main features of the language (Sect. 2) we describe the compilation process (Sect. 3) including the selection of biological components and we study the concrete applications of **Gubs**.

## Language Description

### Presentation

**Fig. 1:**
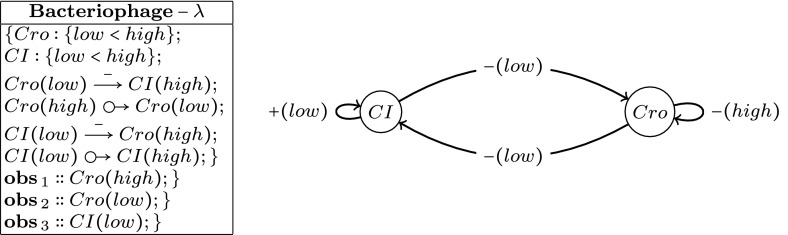
This example introduces the main features of GUBS language applied to a regulatory network of the $\lambda$-phage governing the lysis–lytic cycle and involving Cro and Ci genes. The program is structured as follows: first the relations on attributes are defined, then the regulations are translated to causal relations, and finally the observers are added. Notice, that the inhibition corresponds here to macro relations *i.e.*, $Cro(low)\mathop {\longrightarrow }\limits ^{-}CI(high) =Cro(low)\,{\odot \!\!\;\!\! \rightarrow }\bar{CI(high)};\bar{Cro(low)}$  *CI*(*high*)

The **Gubs** language allows for the description of the behaviour of a biological system validated by a set of observations. In some extend, a **Gubs** program collects the key-observation of an experiment, and ignore those unknown or out of the programming scope. In the following, we will base our examples on the **Gubs** program of Fig. 1 corresponding to lysis/lysogeny genetic regulatory network of bacteriophage lambda (Thieffry and Thomas 1995). The network represents a switch based on two genes where each genes inhibits the other leading to two distinct equilibria.

### Syntax

The elements of a **Gubs** program are defined as follows:

#### Agents and Attributes

*Agents* represent biological elements of the system. They are the atomic elements of the language. In Fig. 1, agents are biological elements such as *Cro* or *CI*

*Constants and variables***Gubs** distinguishes two types of agents: *constants* and *variables*. Constant designates predefined element in a real corpus of knowledge such as a particular gene (*e.g.*, $\textit{Cro}$ in the example); and the variables represent an abstraction of the predefined objects. For example, a variable $g_1$ may qualify a class of gene. By syntactical convention, a constant starts with an Upper case letter and variable with a lower case.

*State of an agent* The different observable states of agents will define their different possible behaviours. These states are associated to the capabilities of actions on the states of other agents. By default, an agent has two abstract states: present or absent, which may correspond to the active or inactive state of a gene for example. The notion of activity is related to the influence capability of an agent in the system. In Fig. 1, the rules consider the active and inactive agents. By convention, the specification of the presence is described with its name (*i.e.*, $\textit{Cro}$) , and begin absent as a negation (*i.e.*, $\overline{\textit{Cro}}$).

*Attributes.* However, restricting the state of a biological agent to the presence or the absence of the agent appears too restrictive to finely describe the intended functionality. Indeed the variation of the concentration of an agent leads to the modification of its interaction with the other agents. In order to account the variation of its activity at different levels, we use the *attribute* characterizing different relevant states symbolically. In Fig. 1, attributes correspond to the variables in parenthesis, for example *low* in *Cro*(*low*). Notice that attributes are defined in the first lines of the program: $Cro:\{low<high\}$.

The biological meaning of the attributes is a matter of convention depending of the target device (*e.g.*, protein reaction, gene network). For example, the activity of gene regulation may correspond to the observation of different thresholds in the RNA concentration inducing different regulatory activities (Bernot et al. 2007). If we identify three regulation activities for a gene *G*, the state of this gene will correspond to three different attributes $\{\textit{Low}, \textit{Mid}, \textit{High} \}$. In order to describe the links between multiple attributes of the same agent, we define two types of relations between attributes qualifying their relative capacity: the order relation, $\prec$, signifies “has less capacity than” meaning that the control capacity at a given state symbolized by an attribute is strictly included in another state symbolized by an attribute greater of the former; and the inequality, $/\!\!\!\!\!\approx$, signifies “has a different capacity than” meaning that the control activity is totally disjoint for any pair of unequal attributes.

*Numerical attributes.* Symbolical attributes are completed by numerical one providing a quantification of the control activity. For example, they may define a concentration measure such as *LacI*(0.3) or a range of values, such as *LaCI*[0.1, 0.4] meaning that the agent is active in this concentration range.

#### Causal Relation and Observation Points

Basically, a causal relation represents the control of an agent over another. In Fig. 1, causal relations between agents are defined as: $\mathop {\longrightarrow }\limits ^{-}$ in $Cro(low)\mathop {\longrightarrow }\limits ^{-}CI(high)$, or  in *Cro*(*high*) *Cro*(*low*).

An historical definition of causality proposed by Hume (1739), is formulated in terms of regularity on events: “[we must define] a cause as an object, followed by another, and where all the similar objects to the first are followed by similar objects to the second”. Although this definition appropriately characterizes the notion of control, the openness of the system should account the actions of the environment that may alter the causal relation chain. For example, an activation $G_1\mathop {\longrightarrow }\limits ^{+}G_2$ may be contradicted by an existing inhibition $G_3\mathop {\longrightarrow }\limits ^{-}G_2$ on the same target gene $G_2$. Although $G_1$ is active, it is possible that $G_2$ is not active because its action is preempted by the action of $G_3$ having stronger strength than $G_1$. Thus, following Hume definition, any planned causal relation could be interrupted unexpectedly by an external event. To circumvent this problem, we semantically define the causal relation in a counterfactual form (Lewis 2000) from the effect: “if the effect is observed, the causal relation is effective”.

The definition of a causal relation will be refined to address the scheduling constraints between cause and effect to be able to more finely select the components corresponding expected behaviour. The causal relation primitives are defined as follows (Basso-Blandin and Delaplace 2013):

- *c**e*: if *e* occurs then *c* has occurred in a recent past.
- $c\,{\odot \!\!\;\!\! \rightarrow }e$: si *e* occurs then *c* has occurred in a recent past and is still present in this moment.
- $c\,{\oplus \!\!\;\!\! \rightarrow }e$: si *e* occurs then, either *e* has occurred in a recent past or else *e* hasn’t occurred in a recent past and *c* then necessarily appeared.

In the sequel, we define the strong inhibition denoted $$g_1 \mathop {\longrightarrow }\limits ^{-}g_2$$ as an inhibition expressed by the program $$\overline{g}_1 {\odot \!\!\;\!\! \rightarrow }g_2, g_1$$$$\overline{g}_2$$.

We also define the strong activation denoted $$g_1 \mathop {\longrightarrow }\limits ^{+}g_2$$ as an activation expressed by the program $$g_1 {\odot \!\!\;\!\! \rightarrow }g_2, \overline{g}_1$$$$\overline{g}_2$$

*Observation spots.* To define the behaviours that we expect to observe in a program, we define the notion of *observation spots*. The observation spots describe the set of expected observations along an experimental trace resulting of a device assessment. Observation points are used to determine effects that must necessarily be fulfilled. In Fig. 1, observation spots are defined for *Cro*(*high*), *Cro*(*low*) and *CI*(*low*) by $\mathbf{obs }\,_1:: Cro(high)$, $\mathbf{obs }\,_2:: Cro(low)$ and $\mathbf{obs }\,_3:: CI(low)$.

#### Compartments and Environments

*Contexts.* In order to describe interactions of the program with the environment, we introduce here the notion of *context*. This notion allows us to clearly differentiate the interactions of the **Gubs** program from those external to the system described by the program. Precisely, a context refers to a stimulus acting on the system that can be either environmental conditions and external signalisation. The application of a *k* context to a set of causal relations *d* is written [*k*]*d* where *k* is an agent (*i.e.*, variable or constant) meaning that the causal relations belonging to *d* are triggered only if the *k* context is present.

*Compartments.* Finally, a **Gubs** program is a set of attributes definition, observation spots and causal relations encapsulated into contexts. In order to describe the spatial organization of the biological system, we introduce a last element in the language, *compartments*. A compartment encapsulates a set of causal relations, making them local. For example, $$C\{ g_1$$$$g_2 \}$$ describe a normal dependence relation in the *C* compartment.

### Interpretation

The result of the compilation is an assembly of components where the behaviour of the generated biological system must comply to the behavioural properties described in the program. In this section, we informally describe the semantics of **Gubs**. Technically, the denotational semantics of **Gubs** is based on multi-modal hybrid logic(HL) translating a **Gubs** program into a formula (Adrien 2014).

Validation of properties is primarily based on a set of experiments measuring the evolution of physical quantities related to agents that qualifies the states of agents. A **Gubs** program describing a symbolic abstraction of these quantities, we assume it is possible to extract a *symbolic trace* characterizing this evolution. This leads to establish a correspondence between physical quantities and attributes. A trace is a sequence, $(T_t)_{1 \le t \le m}$ where each $T_t$ corresponds to an event composed by the state of the agents symbolised by its attributes at each instant. For example, the changes in the concentration of $\textit{Low}$ to $\textit{High}$ of an agent *G* defined with three potential attributes $\textit{Low}$, $\textit{Mid}$, $\textit{High}$ can be described by the following trace composed of 6 instants (Eq. 1) where each value is presumably obtained by a periodical measurement of a quantity related to *G* and symbolically translated.

1 $$\begin{aligned} (\mathop {\{ G(Low) \}}\limits _1, \mathop {\{G(Low) \}}\limits _2, \mathop {\{G(Mid)\}}_3, \mathop {\{G(Mid)\}}\limits _4, \mathop {\{G(Mid)\}}\limits _5,&\mathop {\{G(High)\}}\limits _6) \end{aligned}$$

We now address the principle to extract from a trace an *history* defining a timeline–milestone events of the behavior evolution. Actually, all events in a trace are not necessarily relevant to validate the properties described by the program. For example, if we focus on the evolution of a concentration *Low* to *High* to *G*, only three events are relevant to this description: *G*(*Low*) and *G*(*Mid*), and *G*(*High*), irrespective of the intermediate stages of evolution that occur between the both, nor the repetition identical events. We will therefore adopt a different representation called an history from a chronological division of a trace in several *periods*. An history is an intentional division of a trace to highlight the relevant events emphasizing the desired properties of the behaviour of the biological system. Given a trace $(T_t)_{1 \le t \le m}$, and a chronological division an history is defined as a sequence of set of events occurring during each period.

In the previous example, the period division leading to an history corresponding to the expected evolution from *Low* to *High* for *G* is the following discrete time intervals ([1, 2], [3, 5], [6, 6]). The resulting history is:

$$\begin{aligned} (\{ G(Low) \},\{G(Mid)\},\{G(High)\}). \end{aligned}$$

The validation of a synthetic biological function from a **Gubs** program operates on histories. Formally, *An history corresponds to a Kripke* (1963) *model with the topological property of linearity*(*i.e.*, a graph reduced to a single path). The variables in each world (node) correspond to events (agent’s state). Hence, An history “explains” a **Gubs** program if it satisfies the formula resulting from its interpretation. In some cases, an history represents a partial result of the functionalities of the program only and does not satisfy the formula interpreting the program. Hence, to validate the program it is required to gather different histories such that they will form a Kripke model satisfying the formula interpreting the program. Notice that Tableau method can generate these models (Cerrito and Mayer 2011).

**Fig. 2:**
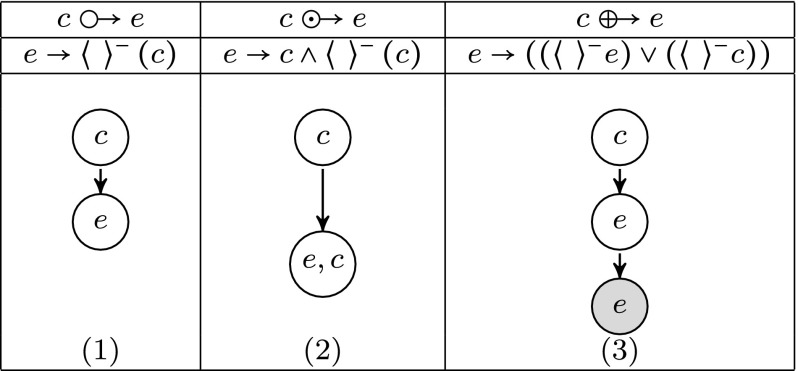
Kripke models corresponding to three types of possible causes. we denote *c* the cause and *e* the effect. In the first case, the model (1) merely imposes *c* to be in a previous world than *e*. In the second case [model (2)], *c* must also appear in the world where *e* is displayed. Finally, in the third case [model (3)], the presence of *e* requires that either *c* appears in the previous world or *e* is displayed in the previous World (*World in gray*)

Figure 2 describes the Kripke models satisfying the different causal relations. Notice that model (1) is a sub-model (limited to white nodes) of model (3). Similarly, model (2) is a special case of model (1) by requiring the presence of the cause in the world of effect. It is worthwhile to point out that a model satisfying a persistent causal relation also satisfies a normal causal relation in turn satisfying remnant causal relation.

## Compilation

The compiler selects and assembles components whose collective behaviour complies to the program specification. Schematically, the compilation principle is based on behaviour pattern matching. The behaviour of each component is also described by a **Gubs** program such that the compilation algorithm collects different component-programs whose assembly “matches” with the behaviour of the programmed function. The generated device $Q=\{Q_i\}$ then behaviourally “covers” the programmed function *P*. Formally, by considering that the interpretation of *P* and *Q* ($[\![{P} ]\!]$,$[\![{Q} ]\!]$) are Hybrid logic formulas, the behavioural inclusion denoted by *P**Q* characterizes the fact that *P* is a logical consequence of *Q* (Definition 1).

### **Definition 1**

(*Behavioural inclusion*) A program *Qbehaviourally includes* another program *P*, if and only if the interpretation of the latter is a logical consequence of the interpretation of the former:where ${\mathcal {M}}$ is a Kripke model and $[\![{P} ]\!],[\![{Q} ]\!]$ are respectively the interpretation of P and Q.

The behavioural inclusion characterizes the condition of correctness for the compilation algorithm to insure that the assembly of components will at least reproduce the behaviour of the designed function. The behavioural inclusion property is defined from the semantics of the program. However, the compilation (the matching) is based on a syntactical comparison between the source and the assembly of component programs. Hence, the compilation process will be defined by a formal system called the *functional synthesis* rules (Table 1) denoted, . It formalizes the operations whereby biological components of a library are selected and assembled to generate a device behaviourally including the programmed function. All the rules of Table 1 preserve the behaviour inclusion (the proofs can be found in Basso-Blandin and Delaplace (2013)). Hence, *Q*${_\sigma}\,P[\sigma ]$ means that the observable assembly of components *Q* is the functional synthesis of *P* that behaviourally includes it under the substitution of $\sigma$ replacing variables by constants (or other variables). It is also worth noticing that the satisfiability of the formula interpreting *Q* is checked to prevent the incompatible assembly revealed here by a false formula. This property is called the observability of *Q* ($\mathbf{obs }\,( Q[\sigma ])$ in Table 1) informally meaning that the assembly does not produce an infeasible behaviour.

**Table 1:**
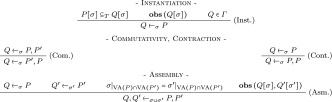
Functional synthesis rules *VA*(*P*) stands for the set of variables of the program and ${\sigma }|_{V}$ is the restriction of the substitution on a set of variables *V*. $\Gamma$ is a set of components representing the library. $P \subseteq _{T} Q$ denotes the textual inclusion of *P* in *Q* *i.e.*, $Q= (Q_1,P,Q_2)$ where $Q_1$ or $Q_2$ may be an empty program

Rule (Inst.) describes the fact that an observable instance of a part of a component in the library is functionally synthesized. Rule (Com.) expresses the commutativity of the assembly. Rule (Cont.) contracts the redundant formulation of programs. Finally, Rule (Asm.) details the conditions for an assembly of two components, each representing a functional synthesis of a part of the designed function. Another set of rules (Adrien 2014) not presented here defines the alternate possibilities to express similar behaviours in order to find components if no components in the database fit to the matching.

The algorithm derived from the formal system can be assimilated to a general unification algorithm (Knight 1989) called the *ACI-unification* (Baader and Büttner 1988; Baader and Snyder 2001) (Associative, Commutative, Idempotent). For the unification, causal rules correspond to normalized terms to unify and a program to a conjunction of terms.

### Compilation Algorithm

ACI-Unification is an NP-Complete problem and the algorithm explores all the possibilities until an unification is found or fail (Baader and Büttner 1988; Baader and Snyder 2001). To improve the algorithm, we account the specificity of Gubs language. The performance of the algorithms actually depends on the size of the database where the components are stored. For a small set of components, the ACI-unification algorithm selects a subset of components behaviourally covering the initial program *P* in the whole database. However, for large database, the unification may be too time-consuming to be effective. In this case, the unification will operate on a part of it. The issue is to appropriately select of a subset of components behaviourally covering *P*. The selection is achieved by a *directed* evolutionary algorithm where the individuals represent a possible subset of components. By directed we mean that we proceed to a pre-selection, checking whether each individual (subset of components) will not obviously cause the unification failure.

Hence, the functional synthesis algorithm is structured in two stages: the ACI-unification algorithm and the directed evolutionary algorithm. An individual represents a subset of components. The best individuals have the minimal number of unified components and maximize the number of unified rules in the program.

Based on ACI unification, the functional synthesis takes benefit of the specificity of the language to heuristically improve the selection of subset of components forming the individuals of a population. The heuristics implies to define a relation between causal relations of *P* and the components $Q_j$ of a database. The relation accounts the structure of the causal rule and the nature of the agents. *P* is assumed to be a set of causes $P=\{p_i\}_i$. The relation qualifies a *local causal association* of *P* where each cause of *P* can be associated to a cause of a subset of components $Q=\bigcup _j Q_j$ without accounting the interplays between the unification of different causal rules. If the causes $p_j$ is not related to some $Q_j$s then the ACI-unification surely fails.

**Fig. 3:**
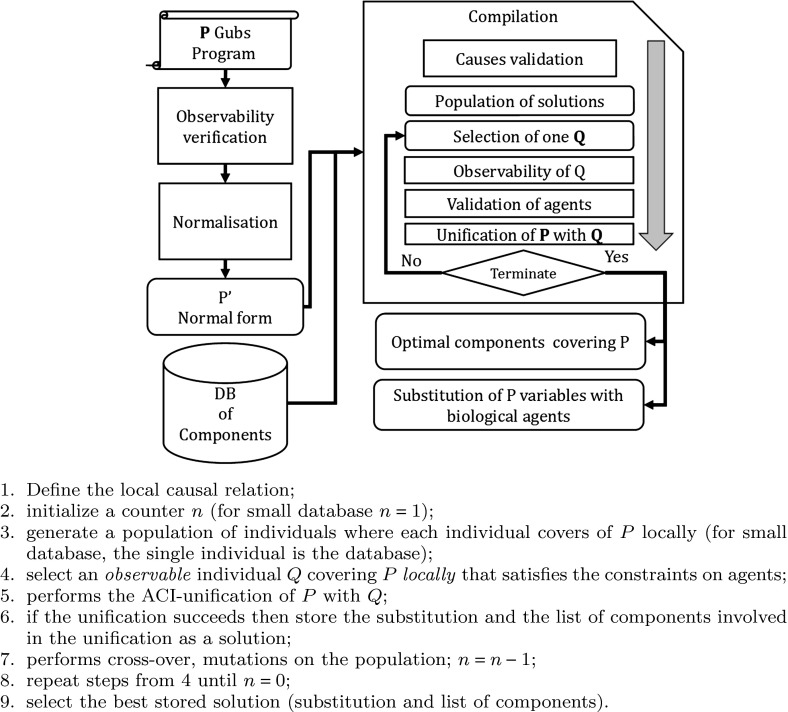
Compilation process

*Constraints on causal relation.* A causal rule $p_i$ is related to a component $Q_j$ if and only if there exists a causal rule $q_j$ of $Q_j$ such that:

1. The type of $q_j$ is the same as $p_i$.
2. All constants of $p_i$ are present in $q_j$ in there respective side of the causal relation(cause or effect).
3. The cardinality of the both set of agents representing the cause and the effect of $q_j$ is greater or equal to the cardinality of the respective set in $p_i$.
4. Each agent of $p_i$ has a counterpart in $q_j$, with the same order on their attributes.
5. For each agent of $p_i$, the number of attributes is lower or equal to the agent counter part in $q_j$.

Once a relation between the causal relations of *P* and those of the components is established, the algorithm selects a subset of components such that there exists at least one causal relation $q_j$ fulfilling the previous constraints for each $p_i$ of *P*. Notice that, different components $Q_j$ of *Q* can cover the same causal rule $p_i$ of *P*. Next, before performing the ACI-unification algorithm between an individual *Q* and *P*, we validate some necessary conditions on the agents.

*Agents constraints.* The agents constraints are the following:

1. The number of occurs of a constant in *P* must be lower or equal to the number of occurs of the same constant in *Q*.
2. For each variable *v* of *P*, there exists at least one constant of *Q* that does not belong to the constants of *P* whose number of occur is greater or equal to the number of occurs of *v* in *P*. Besides the order on the attributes is the same and the number of attributes of the constant is greater the number of attributes of the analysed variable *v*.

The constraints on agents are refined by considering the side of the occur of agents on a causal relation, either as a cause or effect. The main steps of the behavioural matching algorithm are defined in Fig. 3.

### Example: Compilation of the Repressilator

**Fig. 4:**
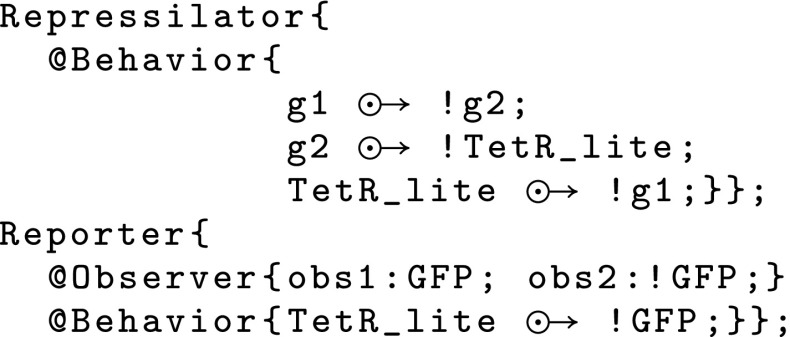
GUBS program of the repressilator

In this section, we sketch the application of the algorithm to the Repressilator (Elowitz and Leibler 2000) example (Fig. 4).

**Fig. 5:**
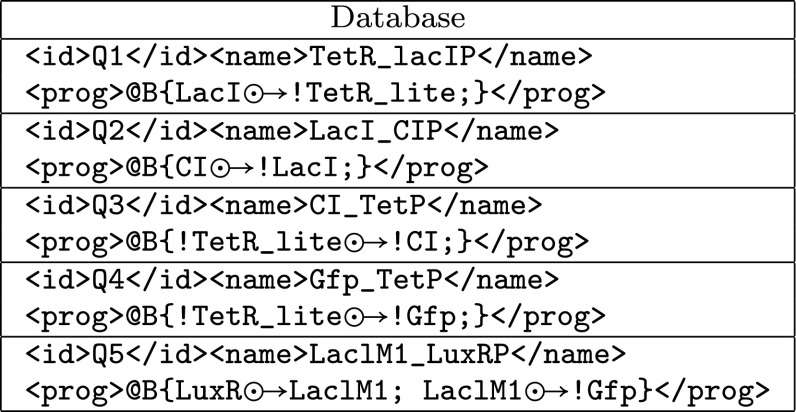
Component database

The Repressilator circuit is one of the first synthetic system leading to oscillation of fluorescent protein (GFP) monitored by a circuit of three genes, each one inhibiting another. To simplify the presentation we apply the algorithm on a piece of the database used for the effective compilation (Fig. 5). In the sequel,the causal relations are labelled by $p_\star$ as follows: $p _1 :$g1${\odot \!\!\;\!\! \rightarrow }$!g2, $p _2 :$g2${\odot \!\!\;\!\! \rightarrow }$!TetR_lite, $p _3 :$TetR_lite${\odot \!\!\;\!\! \rightarrow }$!g1.

1. By application of the first step of the algorithm, we define the relation that locally associate each $p_i, i \in \{1,2,3\}$ to $Q_j$s.
  $p_1$ is locally associated to all the components
  $p_2$ is locally associated to $Q_1$ due to the *TetR_lite* in the effect side
  and finally, $p_3$ is locally associated to $Q_3$ and $Q_4$.
2. Thus, the following population of individuals is generated:
  $$\begin{aligned} \{(Q_1,Q_1,Q_3); (Q_2,Q_1,Q_3); (Q_3,Q_1,Q_3); (Q_4,Q_1,Q_3); (Q_5,Q_1,Q_3);\\ (Q_1,Q_1,Q_4); (Q_2,Q_1,Q_4); (Q_3,Q_1,Q_4); (Q_4,Q_1,Q_4); (Q_5,Q_1,Q_4)\} \end{aligned}$$
3. By application of the agent constraints we have:
  Due to the second rule applied to *g*2, we conclude that $(Q_1,Q_1,Q_3)$ and $(Q_1,Q_1,Q_4)$ cannot cover *P* because *TetR_lite* is used in *P* so it cannot be a substitution of *g*2.
  As for *g*2, $(Q_3,Q_1,Q_3)$, $(Q_4,Q_1,Q_3)$, $(Q_3,Q_1,Q_4)$, $(Q_4,Q_1,Q_4)$ cannot cover *P* because *TetR_lite* is used in *P* so it cannot be substituted to *g*1.
  The unification of $(Q_5,Q_1,Q_3)$ fails because no constants other than *TetR_lite* appear more than once.
  Similarly the unification of $(Q_5,Q_1,Q_4)$ fails *P* because no constants other than *TetR_lite* appear once in a cause and once in an effect.
4. Hence, $(Q_2,Q_1,Q_3)$ and $(Q_2,Q_1,Q_4)$ constitute the remaining choices.
5. The next step of the algorithm consists in the substitution of each variable to constant:
  With this step, using $(Q_2,Q_1,Q_4)$, *g*2 can be substituted to *LacI* but *g*1 cannot be unified.
  So the remaining solution is $(Q_2,Q_1,Q_3)$ where *g*2 is substituted with *LacI* and *g*1 with *CI*.
6. The final solution $(Q_2,Q_1,Q_3)$ corresponds to the biological description of the Repressilator.

Here, due to the unification properties, only one solution is possible, but, in most of the case, several solutions are available. In this case, the potential solutions are ordered with respect to fitness properties.

### Benchmarks and Numerical Results

**Fig. 6:**
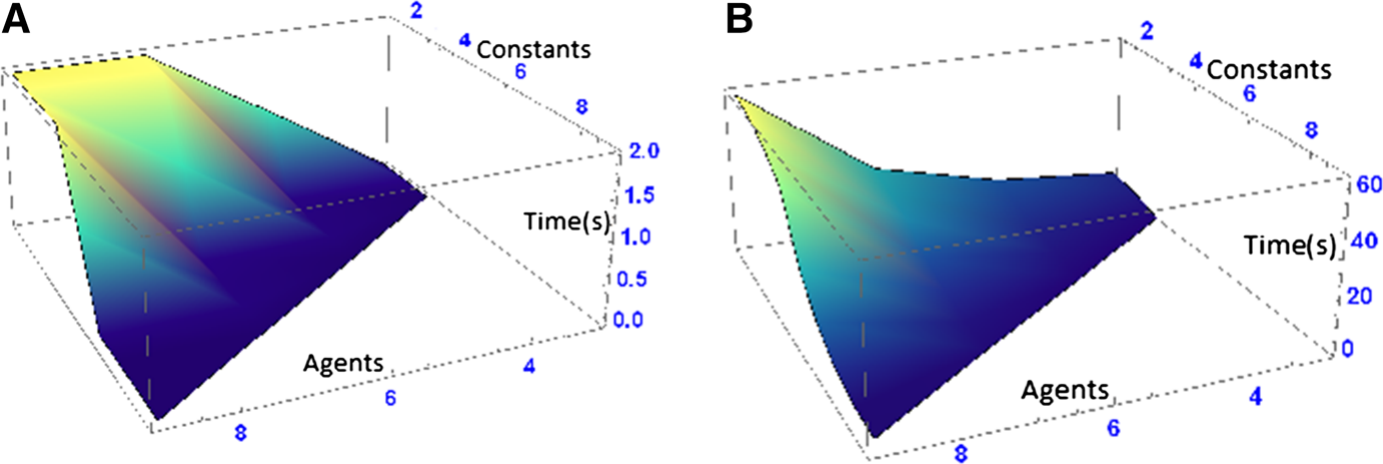
The first curve represent the compilation time evolution (in s) for a 10 causal relations sized program and the second for a 25 causal relations sized program

In order to empirically validate the functional synthesis algorithm, we executed **Ggc** on several data sets. Those data sets are based on databases containing randomly generated 100 components. In order to insure a solution for the tested program and the fact that the compiling time-out is due to the lack of time finding a solution, each program is generated by selecting a subset of components in the database. Execution time on the following curves correspond to program containing respectively 10 and 25 causal relations. In order to show the impact of the number of agents and constants on the compiling time, those numbers evolve from 1 to 10 on each program (Fig. 6).

Those curves confirm the theoretical hypothesis. In fact, we observe that the time evolve exponentially function of the number of variables and not of the agents because constants restrict the possibility of choices. In conclusion, the efficiency of the compilation directly depends on the number of constants corresponding to the known agents.

## Conclusion

In this article, we have described the main features of **Gubs**, a language for synthetic biology based on a behavioural description of the designed biological function. The compilation principle relies on the covering of the behaviour of a programmed function by the behaviour of a collection of components. The behavioural covering corresponds to a matching between component specification and the programmed function specification. The compiler combines the ACI-unification algorithm with a directed evolutionary algorithm enabling to analyse large biological database. We have demonstrated the proof-of-concept of the compilation with a prototype applied on some realistic examples. GGC is implemented in Ocaml, using XML files for the database (the platform is freely available in open source on Basso-Blandin et al. (2015)). GGC has been tested on multiple randomly generated examples and databases (Adrien 2014).

In the future, we could imagine that the design in synthetic biology will require different programming layouts based on different language paradigms structured in a tower of languages and addressing different levels of integration in biology. From a language describing collective operations on cell colonies (Giavitto et al. 2005; Beal et al. 2011) the program will be translated into different intermediate representations to end by a structural low level description programmed in a grammar based language (Cai et al. 2010) of genome sequences. In this tower, the Gubs language occupies the intermediary level dedicated to cell entity behavioural programming. The tower could be completed by methods ensuring the safety of the generated system by a verification of the non-toxicity and simulation for accurately assessing its performance. The integration of these methods could rely on the realization of a connection with existing tools based on a translation of a **Gubs** program into formalism dedicated to toxicity checking as in Giusto et al. (2014) and languages for simulation such as Kappa (Danos et al. 2010) or Biocham (Calzone et al. 2006).

## Footnotes

1 *E.g.*, $Gen(a + b) = Gen(a) \oplus Gen(b)$ where $\oplus$ stands for the calculus of $+$.
